# Apoptosis factors caspases and p53 and their impact in chronic rhinosinusitis with nasal polyposis

**DOI:** 10.1186/2045-7022-3-S2-O20

**Published:** 2013-07-16

**Authors:** Fabiana Valera, Daniel S Küpper, Rafael Malinsky, Cristiane Milanezi, João S Silva, Edwin Tamashiro, Wilma T Anselmo-Lima

**Affiliations:** 1 School of Medicine of Ribeirão Preto - University of São Paulo, Ribeirão Preto, Brazil; 2 School of Medicine of Ribeirão Preto - University of São Paulo, ENT Division, Ribeirão Preto, Brazil; 3 School of Medicine of Ribeirão Preto - University of São Paulo, Biochemistry Department, Ribeirão Preto, Brazil

## Background

The inflammatory process in chronic rhinosinusitis with nasal polyps (CRSwNP) has been extensively studied. However, little is known about the influence of cell death in this disease. Thus, the molecular assessment of mechanisms involved in apoptosis might shed light on the pathogenesis of CRSwNP.

## Objectives

To evaluate the gene expression of different apoptotic factors in patients with nasal polyps and control patients.

## Methods

The mRNA expression of the apoptosis mediators caspases 3, 7, and 9, and p53 protein was analyzed using qRT-PCR in 25 nasal polyps and 18 control samples.

## Results

We observed significantly lower expression of p53 and caspases 3 and 9 genes in patients with CRSwNP compared to the controls, while caspase 7 expression was similarly expressed in both groups.

## Conclusion

The reduced expression of these apoptosis factors in CRSwNP could be related to higher proliferation and perpetuation of inflammatory cells hindering the control of the disease. A better understanding of the possible influence of apoptosis factors on CRSwNP could be a rationale for future therapies.

